# Acute fasciolosis in an alpaca: a case report

**DOI:** 10.1186/s12917-021-02921-x

**Published:** 2021-06-10

**Authors:** C. J. Hayes, P. J. O’Brien, A. Wolfe, S. Hoey, C. Chandler, V. Rhodes, C. I. Carty, I. M. Piras, E. G. Ryan

**Affiliations:** 1 Department of Agriculture Food and the Marine, Cork Regional Veterinary Laboratory, 53 Model Farm Road, Cork, T12YH6E Ireland; 2 University College Dublin, Belfield, Dublin 4, D04W6F6 Ireland; 3 Vetcare, Gallowshill, Athy, Co., Kildare, R14KH33 Ireland

**Keywords:** Alpaca, New World camelid, Acute fasciolosis, Liver fluke

## Abstract

**Background:**

The popularity of new world camelids, particularly alpacas, is growing rapidly in Ireland, presenting a clinical challenge to veterinary practitioners who may not have worked with these species previously. To the authors’ knowledge, the clinical course of a case of acute fasciolosis in an alpaca has not previously been reported, and fasciolosis has not been reported at all in alpacas in Ireland, making this case report a valuable addition to the current literature.

**Case presentation:**

A three-year-old male castrated huacaya alpaca was admitted to UCD Veterinary Hospital with a two-day history of colic and tenesmus. He had been treated with albendazole, dexamethasone and potentiated amoxycillin by the referring veterinary practitioner with no response. On initial clinical exam, sensitivity to abdominal palpation was the only abnormality. However, the alpaca proceeded to show abnormal lying positions, tenesmus and reduced faecal output over the next 24 h. A general blood panel demonstrated moderate anaemia, marked hyperglobulinaemia and moderately increased hepatocellular and hepatobiliary enzyme activity. Abdominal radiography revealed enlargement of the first forestomach compartment without evidence of gastrointestinal obstruction or peritonitis. An abdominal ultrasound exam revealed an elongated, heterogenous mass in the caudoventral abdomen that appeared to be contiguous with the liver. FNA of this mass revealed that it was in fact a liver lobe with biliary stasis and inflammation. Faecal sedimentation demonstrated *Fasciola hepatica* eggs. In spite of treatment with triclabendazole and supportive treatment including blood transfusion, the alpaca’s condition continued to deteriorate and he was euthanised. On post-mortem exam, acute fasciolosis was diagnosed.

**Conclusions:**

The clinical presentation and course of a case of acute fasciolosis in an individual alpaca is described, including the results of a range of diagnostic tests that were carried out. The final diagnosis is supported by a description of post-mortem findings. This information will serve as a resource for veterinary practitioners involved in the diagnosis and treatment of similar cases.

## Background

Keeping of new world camelids, particularly alpacas, is becoming increasingly common in Ireland. Records from University College Dublin (UCD) Veterinary Hospital demonstrate that in the 7 years preceding December 2018 there were five camelid cases admitted to the hospital, compared to 17 cases in the 18 months from then until July 2020. Yet there is a distinct lack of available resources on the topic of new world camelid medicine, with only five relevant veterinary medical textbooks and one peer reviewed journal listed in searches of the UCD library catalogue, Pubmed and Google Books. The importance of resources in medical decision making is recognised, with decisions based on incomplete anecdotal knowledge having the potential to lead to errors [1]. It is imperative that a bank of reliable, accessible, peer-reviewed literature is built up in order to disseminate the available knowledge on camelid medicine and ultimately promote the health of these species. This case report will contribute to this evidence-based medicine approach.

The aim of this case study is to describe the clinical presentation and course of an individual case of acute fasciolosis in an alpaca, including the results of a range of diagnostic tests. As far as the authors are aware, this has not previously been reported in the peer-reviewed literature. Both acute and chronic forms of fasciolosis are reported to occur in camelids, the chronic form more commonly [2]. *Fasciola hepatica* is reported to cause clinical disease at the herd level in llamas in the UK [3] and in alpacas in Peru at altitudes less than 4000 m above sea level [4]. It has also been found in individuals of both species on post-mortem exam in Switzerland [5] and the US [6]. Given this widespread distribution, in situations where alpacas are kept for farming and commercial purposes, fasciolosis will have important economic implications due to the potential for mortality, as well as loss of production and treatment costs [3]. To the authors’ knowledge, fasciolosis has not yet been reported in camelids in Ireland. Furthermore, colic has not been reported as a primary presenting sign of acute fasciolosis. This novel case demonstrates that it should be a differential for any grazing or previously grazed alpaca presenting with abdominal pain, particularly if liver enzyme activity is raised, and even if previous flukicide treatment is reported in the history.

## Case presentation

### Demographic details

A three-year-old male castrated huacaya alpaca weighing 81 kg presented in late November 2019 to UCD Veterinary Hospital along with a healthy companion alpaca.

### Medical history

The alpaca had a two-day history of colic signs (rolling and vocalisation), tenesmus and reduced faecal output. He had been seen by the referring veterinary practitioner, who treated him with albendazole and dexamethasone and started him on a course of potentiated amoxycillin, but he failed to respond to this treatment. Prior to that, he had most recently received antiparasitic treatment in the form of albendazole in early September. He received annual vaccinations against clostridial disease. He was kept outdoors with a second alpaca and grazed alongside sheep.

### Symptoms and signs

On initial clinical exam, the alpaca was quiet, alert and responsive. Body condition was moderate to good. His respiratory rate was increased at 36 breaths per minute (normal range: 10–30), likely due to the stress of travelling and restraint, but his heart rate (88 beats per minute, normal range: 60–90 beats per minute) and temperature (38.2 °C, normal range: 37.5–38.9 °C) were within normal limits [2]. Mucous membranes were a pale pink colour. First compartment (C1) contractions were reduced in frequency at one every 2 min. He resented abdominal palpation, particularly in the cranioventral region, signified by a loud grunting sound and attempts to escape restraint. Otherwise, there were no abnormalities identified.

Over the following 24 h of hospitalisation however, the alpaca demonstrated prolonged recumbency, frequently lying in a semi-lateral position with the limbs partially extended rather than cushing with all four legs tucked underneath him. This was assumed to be indicative of abdominal pain. He also displayed intermittent episodes of tenesmus and passed minimal numbers of faecal pellets of a normal colour and consistency. He consumed only a very small amount of hay and was not observed eating the concentrate feed offered.

Blood for haematology (Advia 2102, Siemens, Dublin, Ire) and plasma biochemistry (Atellica CH 930, Siemens, Dublin, Ire) was collected via jugular venipuncture on the day of admittance to the hospital and on the fifth and seventh days of hospitalisation. Reference intervals (RI) for alpacas specific to the UCD clinical pathology lab were not available, so RI described in Cockcroft et al. [7] and Dawson et al. [8] were used to interpret results.

Haematologic analysis (Table 1) indicated moderate, macrocytic, hypochromic anaemia with moderate reticulocytosis. This regenerative anaemia could not be attributed to the accompanying, moderate hypophosphatemia found with biochemical analysis (Table 2), which was insufficiently severe to cause haemolysis. Nor could it be attributed to mycoplasma infection, which could not be identified on blood smears.

**Table 1:** Haematology results for a 3-year old male huacaya alpaca diagnosed with acute fasciolosis

Parameter^**1**^	Reference interval^**2**^	Day 1	Day 2	Day 4	Day 5	Day 7	Day 8
PCV/Haematocrit (l/L)	0.24–0.36	0.16_b_	0.15_b_	0.16_b_	0.15_b_	0.13_b_	0.13_b_
Haemoglobin (g/L)	104–170	57_b_	np^3^	np	55_b_	51.0_b_	np
RBC (× 10^12^/L)	9.1–13.8	5.02_b_	np	np	4.83_b_	4.38_b_	np
MCV (fl)	21.8–28.9	30.8_a_	np	np	30.4_a_	30.4_a_	np
MCH (pg)	10.6–12.7	11.4	np	np	11.4	11.7	np
MCHC (g/L)	418–496	370_b_	np	np	373_b_	384_b_	np
Reticulocytes (× 10^9^/L)	< 82	317a	np	np	192_a_	239a	np
Reticulocytes (%)	< 1.5	7.4_a_	np	np	4.0_a_	5.5_a_	np
nRBC (×109/L)	0	0	np	np	0.42_a_	0	np
Platelets (×10^9^/L)	220–947	1250_a_	np	np	1341_a_	1351_a_	np
WBC (× 10^9^/L)	7.1–18.6	40.05_a_	np	np	20.75_a_	19.20_a_	np
Neutrophils (× 10^9^/L)	3.5–12.1	31.64_a_	np	np	3.11_b_	6.34	np
Bands (×10^9^/L)	0–0.1	0	np	np	3.74_a_	0.38_a_	np
Lymphocytes (×10^9^/L)	1.5–4.7	1.2_b_	np	np	1.45_b_	6.53a	np
Monocytes (×10^9^/L)	0–0.9	1.6_a_	np	np	4.36_a_	3.65_a_	np
Eosinophils (×10^9^/L)	0.4–4.0	5.61_a_	np	np	8.09_a_	2.30	np

^1^ PCV: packed cell volume, RBC: red blood cell count, MCV: mean cell volume, MCH: mean cell haemoglobin, MCHC: mean cell haemoglobin concentration, nRBC: nucleated red blood cell count, WBC: white blood cell count

^2^ Dawson et al. [8]

^3^ np: not performed

_a_ Above the reference range

_b_ Below the reference range

**Table 2:** Plasma biochemistry results for a 3-year old male huacaya alpaca diagnosed with acute fasciolosis

Parameter^**1**^	Reference interval^**2**^	Day 1	Day 2	Day 4	Day 5	Day 7	Day 8
Total Protein (g/L)	52–65	89.7_a_	88_a_	91_a_	84.7_a_	75.6_a_	80_a_
Albumin (g/L)	30–41	28.1_b_	np^3^	np	27.4_b_	25.3_b_	np
Globulin (g/L)	21–36	61.6_a_	np	np	57.3_a_	50.3_a_	np
Urea (mmol/L)	3.9–10.2	12_a_	np	np	23_a_	19.9_a_	np
Creatinine (μmol/L)	90–140	145_a_	np	np	258_a_	184_a_	np
Glucose (mmol/L)	4.0–6.4	9.51_a_	np	np	11.13_a_	8.50_a_	np
β-OH-butyrate (mmol/L)	0–0.24	0.15	np	np	0.26_a_	0.45a	np
Phosphate (mmol/L)	1.1–2.5	0.50_b_	np	np	np	np	np
Calcium (mmol/L)	2.1–2.5	2.2	np	np	2.20	2.1	np
Magnesium (mmol/L)	0.8–1.0	0.97	np	np	1.00	0.86	np
GGT (U/L)	12–34	191_a_	np	np	250_a_	244_a_	np
Lipase (U/L)	9–17	10	np	np	19_a_	9	np
GLDH (U/L)	5–26	81_a_	np	np	69_a_	64_a_	np
AST (U/L)	160–274	173	np	np	212	222	np
CK (U/L)	29–120	51	np	np	88	70	np

^1^ β-OH-butyrate: β-hydroxy-butyrate, GGT: gamma-glutamyltransferase, GLDH: glutamate dehydrogenase, AST: aspartate aminotransferase, CK: creatine phosphokinase

^2^Cockroft et al., [7]

^3^ np: not performed

_a_ Above the reference range

_b_ Below the reference range

Marked inflammation was indicated by marked leucocytosis due to marked neutrophilia, moderate monocytosis, and mild eosinophilia, along with moderate, reactive thrombocytosis. This was confirmed on the biochemistry panel by marked hyperproteinaemia due to marked hyperglobulinaemia, and mild hypoalbuminaemia. Mild lymphopaenia and moderate hyperglycaemia may be attributable to stress.

There was mild azotaemia with mildly increased urea and creatinine. This may have been pre-renal as eating and drinking were reduced.

Marked hepatopathy was indicated by a marked (6-fold the upper limit of normal[ULN]) increase in gamma-glutamyl transferase (GGT), with a moderate (3-fold ULN) increase in hepatocellular glutamate dehydrogenase (GLDH) activity (Table 2).

Total calcium, beta-hydroxy-butyrate, calcium, magnesium, aspartate aminotransferase (AST), creatine kinase, and pancreatic lipase were within reference range.

**Fig. 1:**
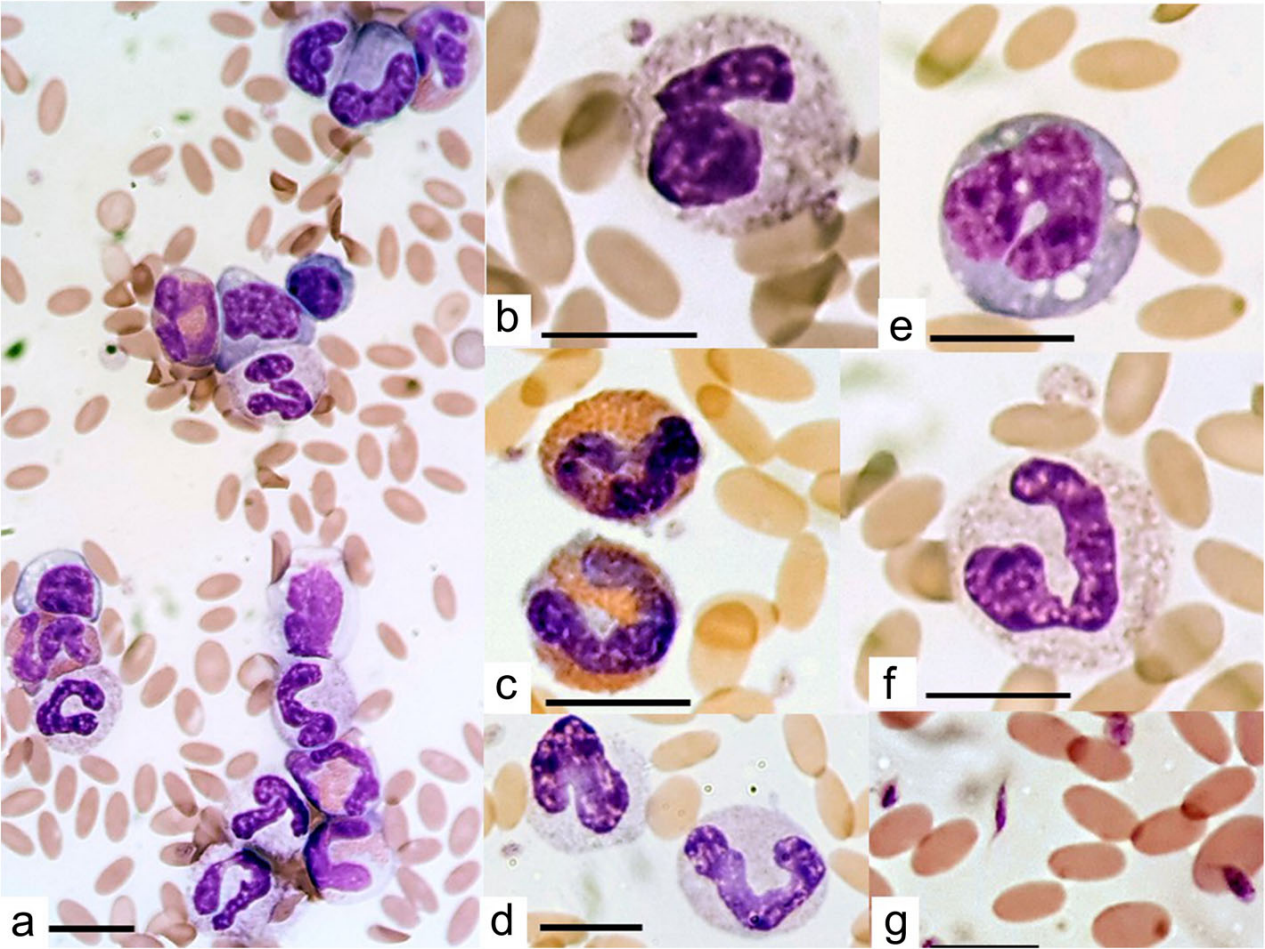
Blood smear taken on day five of hospitalisation of an alpaca diagnosed with acute fasciolosis. Photomicrograph (**a**) demonstrates decreased erythrocyte density, predominance of eosinophils and immature neutrophils. Other photomicrographs demonstrate close-ups of toxic neutrophils (**b**,**f**), eosinophils (**c**), immature neutrophils (**d**), monocytes (**e**) and pleomorphic platelets (**g**). Images taken using Motic BA410 microscope, Motic Moticam 10 (10.0 mp) camera and Motic Images Pluse 2.0 image processing software, all purchased from Motic Europe, Barcelona, Spain. 100 erythrocytes were measured using the Motic calibration cytometer and their mean size was found to be 6.23 μm with a standard deviation of 0.56 μm and range 4.7 to 7.7 μm. Erythrocyte size was normally distributed. A scale bar in the bottom left of each photomicrograph indicates 10 μm

Clinical pathology changes substantially deteriorated from day 1 to day 5. There was a mild deterioration in the anaemia and hypoproteinemia. A poor prognosis was indicated by the development of a degenerative left shift by day 5, with mild neutropaenia, increase in bands to a higher count than segmented neutrophils (degenerative left shift) and mild toxic changes seen in neutrophils on blood smears (Fig. 1). The eosinophilia had worsened to a moderate severity. A mild rubricytosis developed. Also, the severity of the azotaemia had doubled. However, due to the difficulty in collecting a urine sample, renal tubular concentrating ability could not be determined. Hepatobiliary pathology deteriorated with GGT activity having increased by a third, whereas hepatocellular GLDH activity decreased by 15%. Pancreatic lipase had doubled, a mild ketonemia developed, and there was a further, mild increase in blood glucose. Unfortunately, the clinical pathology lab was unable to re-analyse phosphate as it had run out of reagent.

On the seventh day, there were a few further, noteworthy changes in clinical pathology parameters. The anaemia had mildly worsened. However, the leukogram had largely normalised, although there was persistent monocytosis and a mild lymphocytosis had developed. The ketonemia was mild, although ketones had doubled. Pancreatic lipase had normalized.

Faecal flotation using McMaster’s technique was performed on the day of admittance, with no nematode eggs detected. The reduced sensitivity of this technique compared to the modified Stoll’s technique is acknowledged [9]. However, if high counts are expected, as with a burden of gastrointestinal nematodes causing clinical disease, McMaster’s technique can still be appropriate [10]. Faecal sedimentation was not requested on the first day of hospitalisation as, due to the history of recent treatment with albendazole, an adult fluke infestation was not suspected. Faecal flotation was repeated and sedimentation carried out as the combination of anaemia, increased hepatobiliary enzyme activity and rising eosinophilia made *F. hepatica* infestation a more likely differential diagnosis. Again, no nematode eggs were detected. However, *F. hepatica* eggs were present.

Faeces were assessed for the presence of occult blood, consistent with third compartment ulceration (TCU), yielding a negative result. However, due to reduced faecal output, the required 50 g sample could not be provided, reducing the sensitivity of the test. It was also acknowledged that the negative predictive value of the faecal occult blood test for TCU in camelids is questionable [9].

**Fig. 2:**
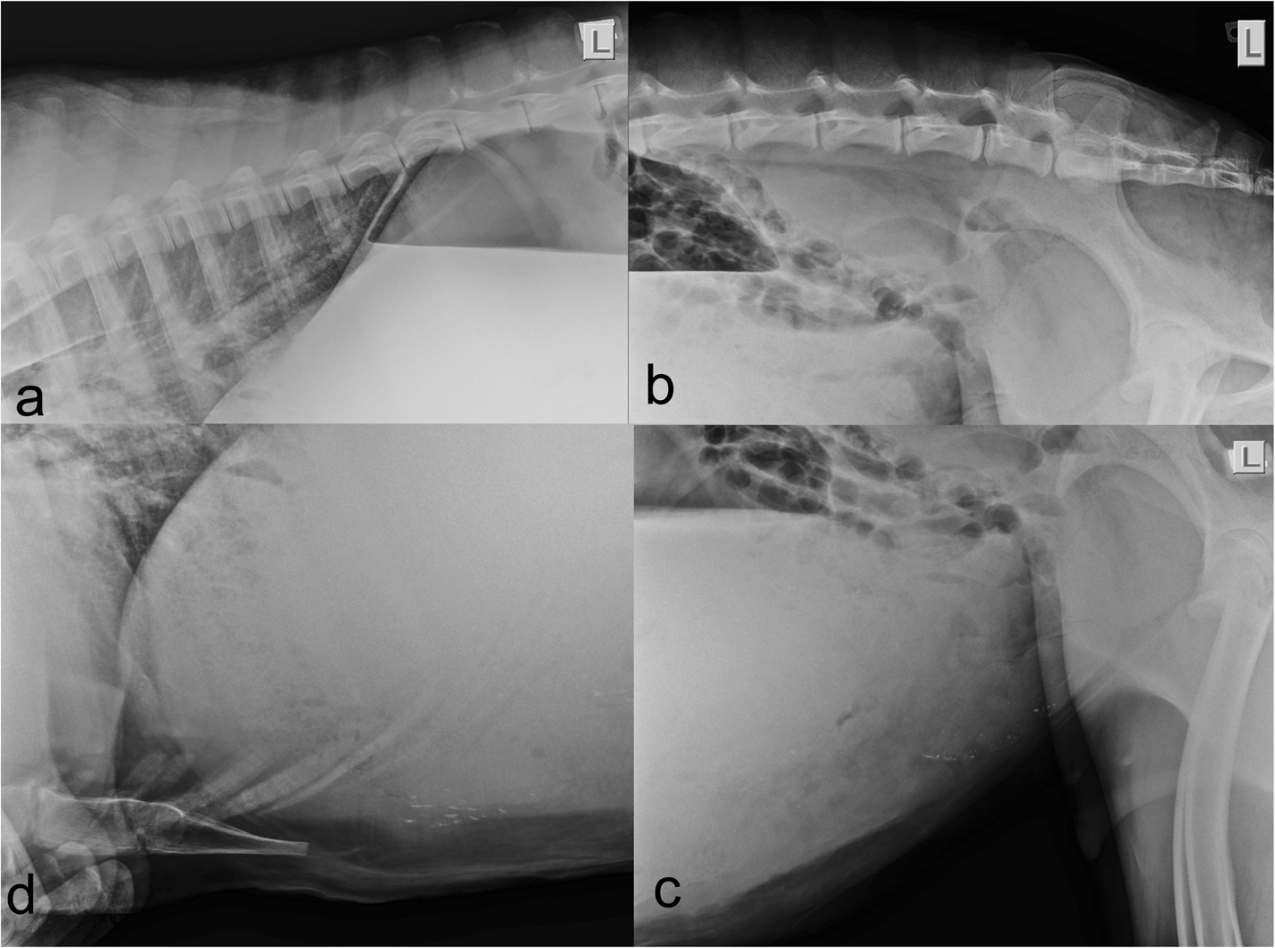
Lateral abdominal radiograph of a three-year old male huacaya alpaca diagnosed with acute fasciolosis. Sub-figure **a** shows the dorsal thorax and abdomen. Sub-figure **b** shows the caudodorsal abdomen. Sub-figure **c** shows the caudoventral abdomen. Sub-figure **d** shows the cranioventral thorax and abdomen

Gastrointestinal obstruction or peritonitis secondary to perforation of a TCU or other source, consistent with the presenting signs of abdominal pain and reduced faecal output, were questioned. Standing, unsedated, lateral abdominal radiographs (Fig. 2) demonstrated a moderate enlargement of C1 and a normal soft tissue-gas opacity interface within the same compartment. Multiple foci of mineral opacity superimposed the ventral aspect of the third compartment. There were no extra-compartmental gas opacities or evidence of poor serosal detail suggestive of a peritoneal effusion. These findings were interpreted as mild dilation of C1, with no evidence of a mechanical obstruction or peritonitis.

**Fig. 3:**
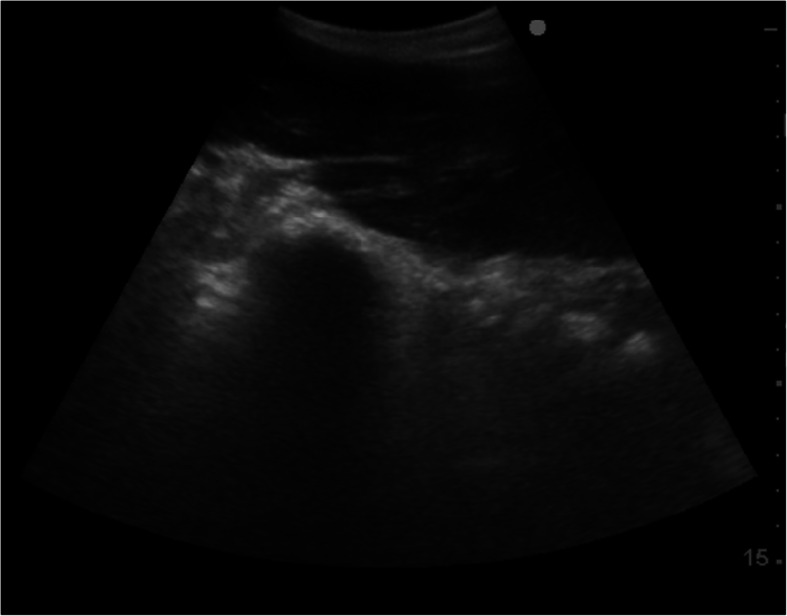
Ultrasound image of a caudoventral abdominal mass identified in an alpaca diagnosed with acute fasciolosis

An abdominal ultrasound examination was performed. The fibre was not clipped, but rather parted at the skin, and surgical spirit was applied directly to the skin of the abdominal body wall. The caudal pleural interface and liver were first imaged through the ninth, tenth and eleventh intercostal windows, with no abnormalities identified. Within each of these spaces, the probe was moved ventrally until the third stomach compartment (C3) could be imaged. No disruption to the integrity of the wall of C3, indicative of TCU, nor any extracompartmental fluid or gas suggestive of perforation of the compartment, could be imaged. Small intestinal loops were imaged caudal to C3. Motility was subjectively reduced. An elongated, irregularly outlined mass was identified in the right ventral abdomen, adjacent to the abdominal wall (Fig. 3). The appearance was similar to hepatic parenchyma but was mildly heterogeneous in echogenicity. When the probe was moved cranially along the mass, it appeared contiguous with the liver, but protruded caudal to the costal arch. In new world camelids, the liver is normally only visible intercostally [11]. The kidneys, intact bladder, spleen and wall of C1 were also imaged, with no abnormalities detected. No excessive free peritoneal fluid was detected during the ultrasound examination.

**Fig. 4:**
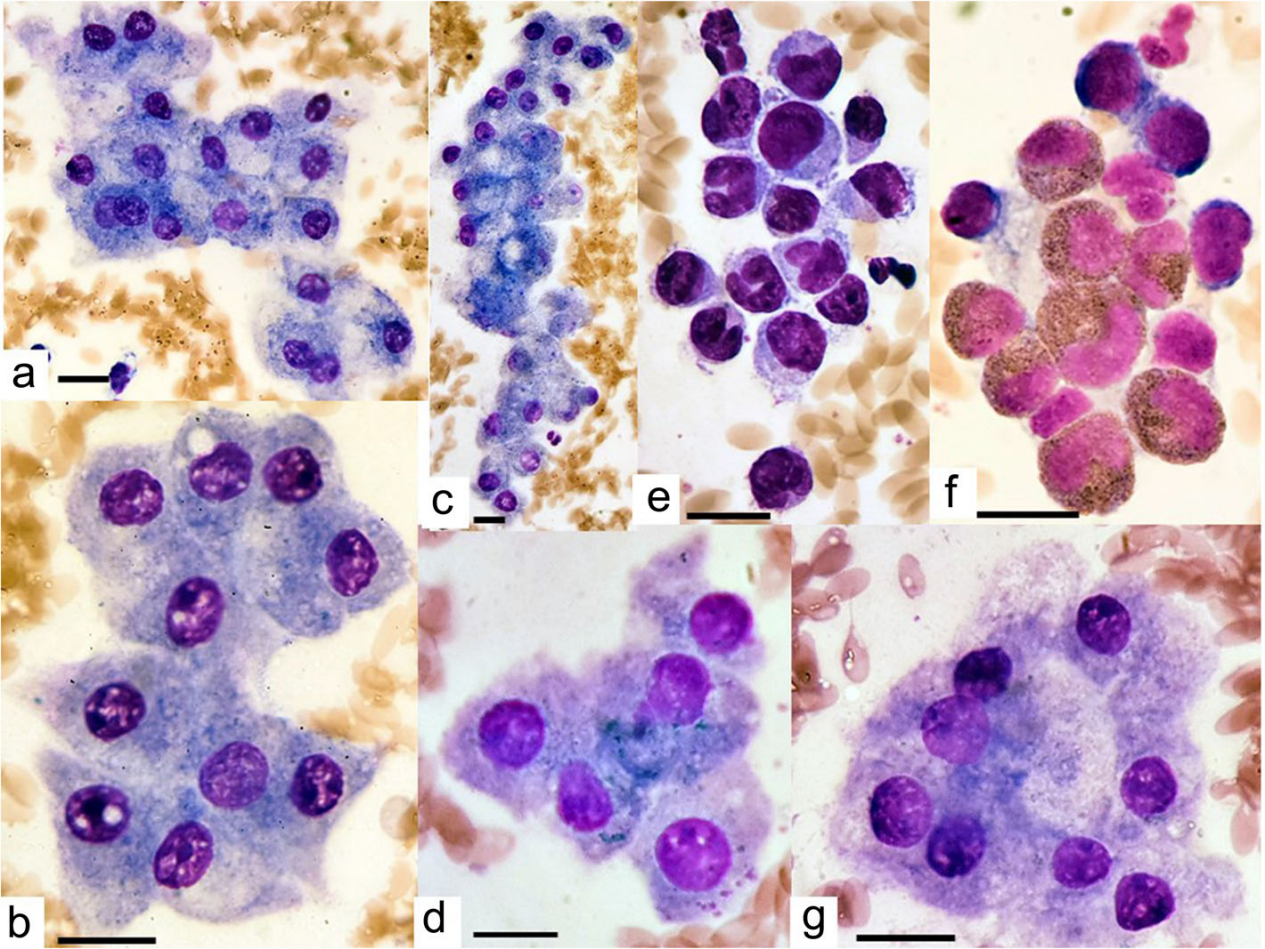
Cytological findings from ultrasound-guided, fine-needle aspiration of an abdominal mass in an alpaca. Photomicrographs **a** to **d** and **g** demonstrate monomorphic hepatocytes with mild vacuolar change (**a**, **c**) cell swelling (**a**, **g**), mild accumulation of tiny, blue-green, granules conistent with bile (**a**, **c**, **d**) and binucleation (**a**). Photomicrographs E and F show inflammation with increased numbers of neutrophils and mononuclear cells (**e**) and eosinophilis (**f**). Images taken using Motic BA410 microscope, Motic Moticam 10 (10.0 mp) camera and Motic Images PLUse 2.0 image processing software, all purchased from Motic Europe, Barcelona, Spain. 100 erythrocytes were measured using the Motic calibration cytometer and their mean size was found to be 6.23 μm with a standard deviation of 0.56 μm and range 4.7 to 7.7 μm. Erythrocyte size was normally distributed. A scale bar in the bottom left of each photomicrograph indicates 10 μm

An ultrasound-guided fine needle aspirate of the abdominal mass was performed. The alpaca was restrained unsedated while cushing and an area of fibre was clipped from the abdominal wall overlying the mass. The skin was prepared with chlorhexidine scrub, followed by surgical spirit. The mass was localised and a 1.5″ 18 g needle attached to a 2 ml syringe introduced alongside the probe, through the body wall and into the mass until it could be imaged within the mass. Negative pressure was applied using the syringe and the needle redirected within the mass. Negative pressure was released and the needle withdrawn. The contents of the needle were then expelled onto a glass slide and smeared with a second slide. The process of fine needle aspiration was repeated twice. The smeared, air-dried samples were submitted for cytological examination. The cytological findings were in fact consistent with cells of hepatic origin, with clusters or sheets of large, epithelioid, monomorphic, polygonal cells with basophilic cytoplasm occasionally containing small amounts of blue-green pigmented granules, consistent with bile (Fig. 4). In the background were large numbers of granulocytes and mononuclear cells. There were no criteria of malignancy. It was concluded that there was mild hepatocellular cholestasis along with mild, mixed granulocytic and mononuclear inflammation. Photomicrographs of the cytological samples were taken using Motic BA410 microscope, Motic Moticam 10 (10.0 megapixels) camera and Motic Images Pluse 2.0 image processing software, all purchased from Motic Europe, Barcelona, Spain.

**Fig. 5:**
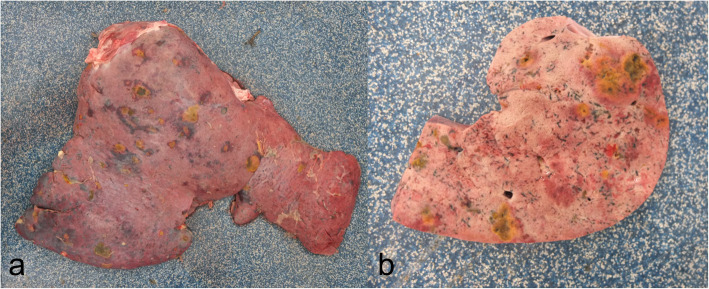
Gross pathological images of the liver of an alpaca diagnosed with acute fasciolosis. Sub-Fig. **a** shows the entire liver from the diaphragmatic surface. Sub-Fig. **b** shows a cut-section through the liver

**Fig. 6:**
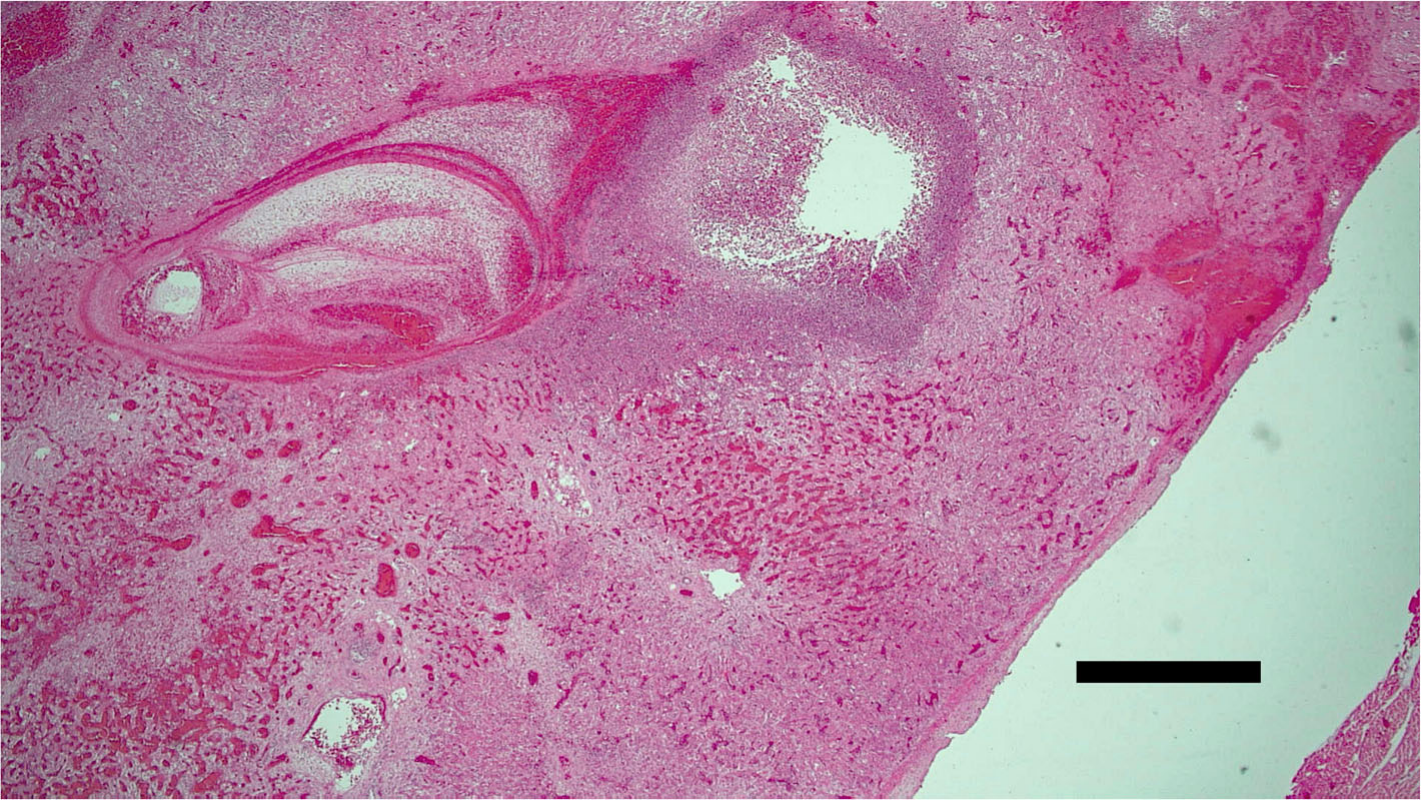
Histological image of the liver from an alpaca diagnosed with acute fasciolosis. Loss of normal hepatic architecture due to parasitic tracts, associated inflammatory changes and haemorrhage are demonstrated. H&E, × 20. Image taken using Olympus BX43 microscope, HD Chrome Exofocus camera and TCapture imaging software. A scale bar in the bottom right of the photomicrograph indicates 1 mm

Following eventual euthanasia, a post-mortem examination was carried out. Overall, the animal was in moderate body condition with moderate subcutaneous and visceral fat reserves. The main pathological findings centred on the liver which was enlarged (6% of body weight) and contained multiple yellow foci of necrosis ranging from 0.3 to 3 cm in diameter which were surrounded by a haemorrhagic rim (Fig. 5). In addition, there were linear pale and red areas of possible fibrosis and haemorrhage scattered throughout the hepatic parenchyma consistent with parasitic migration tracks. Bile ducts were dilated with thickened walls and, on cut-surfaces, contained immature trematodes within their lumens. In addition to the hepatic changes the animal was also suffering from a fibrinous peritonitis and pericarditis, congestion and oedema of the mucosa of C3 and the duodenum, enlargement of the hepatic, mesenteric and tracheobronchial lymph nodes and multifocal petechial haemorrhages in the subcutaneous tissues of the thorax, the muscles of the neck and the pleura. Histologically over 80% of selected liver sections were markedly to severely disrupted by inflammatory changes (Fig. 6). Multifocally there was complete loss of normal architecture which was replaced by massive numbers of inflammatory cells (mostly eosinophils, with fewer lymphocytes and neutrophils) and cellular debris, surrounded by a rim of haemorrhage. Focally there was extensive individualisation and loss of hepatocytes with associated marked disruption of normal cord architecture. Multifocally to coalescing there were markedly increased numbers of mixed inflammatory cells within sinusoids. Multifocally to coalescing there was a moderate to marked proliferation of fibroblasts (fibrosis). Photomicrographs of the histopathological slides were taken using an Olympus BX43 microscope, HD Chrome Exofocus camera and TCapture imaging software.

### Treatment and interventions

As haematology and biochemistry demonstrated inflammatory processes (eg. leucocytosis, degenerative left shift and hyperglobulinaemia) which could have been due to infection, the alpaca was maintained on once daily potentiated amoxycillin (7 mg/kg amoxycillin, 1.7 mg/kg clavulanic acid, intramuscularly) throughout hospitalisation. An intravenous catheter was placed in the jugular vein and he was administered esomeprazole (0.4 mg/kg, intravenously) once daily to treat and prevent TCU. Initially, no non-steroidal anti-inflammatory drug was given due to concerns about TCU as the possible cause of the abdominal pain he presented with. Instead, buprenorphine (0.01 mg/kg) was administered intravenously. This resulted in severe dysphoria, with vocalisation and frenzied activity. Following this, meloxicam was given once daily to provide pain relief (0.25 mg/kg, intravenously). When concerns were raised about chronic or acute fasciolosis, the alpaca was treated with triclabendazole (15 mg/kg, per os).

As his condition began to deteriorate on day eight, he was administered intravenous fluids (Hartmann’s solution, 6 ml/kg/hour) and a blood transfusion was performed. This was collected from the healthy companion alpaca into a 450 ml pre-citrated blood collection bag. Due to lack of cooperation on the part of the donor alpaca, only 300 ml of whole blood could be collected and delivered.

### Outcomes

Initially, the alpaca’s condition improved. He was observed standing easily or cushing rather than lying in a semi-lateral position and eating a small amount of hay and concentrate feed. Unfortunately, on day six of hospitalisation he began to deteriorate. He returned to spending long periods lying in a lateral or semi-lateral position with the limbs partially or fully extended. He became completely anorexic. On day eight, he assumed complete lateral recumbency and was unable to rise, with a heart rate of 128 beats per minute. In comparison to day one, there was an approximate decrease in both haematocrit and total protein by 15%, suggestive of mild haemorrhage.

His condition did not improve, in spite of supportive care, and he was euthanised with an overdose of intravenous barbiturate (100 mg/kg). On post-mortem examination it was determined that acute fasciolosis was the likely cause of death.

The companion alpaca was also treated with triclabendazole (15 mg/kg, per os) and faeces were collected from him for flotation and sedimentation. 50 strongyloid eggs per gram were present, as well as one fluke egg.

Fluke control measures were subsequently discussed with the owner, including chemical prophylaxis, regular faecal egg counts and, if possible, the identification and fencing off of fluke habitats to reduce reliance on fasciolicides.

## Discussion and conclusions

Fasciolosis in Irish alpacas has never been described, to our knowledge. Given that it affects these species in other parts of the world where *F. hepatica* is endemic, it can be assumed that its apparent prevalence will increase as keeping of alpacas gains popularity in this country.

Acute fasciolosis has been reported to result in clinical signs including sudden death, depression or coma, weakness, recumbency, loss of body condition, anorexia, ill thrift, diarrhoea, haemoglobinuria, icterus, haemorrhage, dyspnoea, hepatic encephalopathy and tenesmus [2, 4]. In this case, although the alpaca did present with anorexia and tenesmus, depression and recumbency did not develop until more than 1 week after abnormal clinical signs first developed. The authors are not aware of colic having previously been reported as a primary presenting sign in a case of acute fasciolosis in an alpaca.

Differential diagnoses for an adult male alpaca with a subacute history of anorexia, tenesmus and abdominal pain with reduced faecal output and without diarrhoea include:

- TCU, with or without perforation and associated peritonitis
- Peritonitis from a source other than a TCU e.g. perforation of another part of the gastrointestinal tract
- Gastrointestinal obstruction e.g. impaction, intussusception or strangulation
- Urethral obstruction
- Pain due to stretching of a non-digestive organ capsule e.g. liver enlargement with hepatitis

Tenesmus without diarrhoea or colitis can also be caused by neurological disorders. However, as the alpaca displayed no additional neurological symptoms, these were not considered amongst the differential diagnoses.

TCU is almost impossible to diagnose definitively antemortem, and in fact was not ruled out in this case until post-mortem exam. A faecal occult blood test was negative, but a negative result cannot rule out TCU [9]. There were also no ultrasonographic changes indicative of TCU. Even if TCU had been present, ulceration normally occurs secondary to a stressor such as disease, metabolic strain or changes in the alpaca’s social group or environment [9] and is not normally the primary disease process.

There was no evidence of peritonitis on diagnostic imaging. The peritonitis found on post-mortem examination may not have been severe enough at the time the diagnostic imaging was carried out to be detected. This was also likely to have occurred secondary to the intraperitoneal and systemic inflammation engendered by the severe hepatitis that was present in association with the juvenile fluke infestation, and was not the primary disease process.

Impaction of the first or third stomach compartment can occur in New World camelids, which could initially present, as this case did, with anorexia, tenesmus, abdominal pain and reduced faecal output. However, clinical signs would be expected to progress to a degree of abdominal distension [12], which this alpaca did not develop. There were also no indications of gastric impaction on abdominal imaging, although this cannot definitively rule out an impaction. An animal with an acute intestinal obstruction such as an intussusception, impaction or strangulation would be expected to deteriorate more quickly than occurred in this case, where the clinical picture did not deteriorate significantly until the sixth day of hospitalisation.

Urethral obstruction occurs not infrequently in both castrated and intact male New World camelids of all ages [13]. The tenesmus and fact that the alpaca was never observed passing urine increased the index of suspicion of this differential diagnosis on presentation. However, when repeated plasma biochemistry tests failed to demonstrate azotaemia of severity consistent with urinary tract obstruction, and diagnostic imaging did not indicate enlargement of the bladder or free peritoneal fluid to suggest urinary tract rupture, this differential was considered very unlikely.

The diagnosis of acute fasciolosis was not confirmed until post-mortem examination, highlighting the difficulty in clinical diagnosis of this disease. However, the presence of fluke eggs on faecal sedimentation, as well as the eosinophilia on haematology and raised liver enzyme activity on biochemistry, did make fasciolosis (with or without concurrent TCU, which was not ruled out until post-mortem) the primary differential diagnosis for this case.

Acute fasciolosis in alpacas has been successfully treated with a single oral dose of 15 mg/kg of triclabendazole and moving to low-risk pasture [4]. Oral clorsulon, given twice at 45 to 60 day intervals at a dose of 7 mg/kg is also recommended in camelids, as well as albendazole at a dose of 15 mg/kg [2], although it must be noted that these compounds are only effective against adult fluke. The question arises as to why, in this case, both *F.hepatica* eggs in the faeces and adult fluke in the liver were found when the animal’s history included treatment with albendazole as recently as ten days before euthanasia and post-mortem, which should have been effective against adult fluke. Although apparently not as widespread as resistance to triclabendazole, resistance of *F. hepatica* to albendazole has been reported in sheep in Spain [14], Argentina [15] and possibly in Sweden [16]. Although cases of albendazole resistance have not yet been reported in Ireland or in camelids, this alpaca was grazed alongside sheep and exposure to albendazole-resistant fluke, if they were present, is plausible. The dose of oral albendazole administered just prior to referral was not recorded. However, albendazole toxicity has been reported in alpacas at doses as low as 19 mg/kg, with effects ranging from alopecia to death [17, 18]. This knowledge may result in caution amongst veterinary practitioners when calculating dosages, especially when weight cannot be accurately ascertained, and may inadvertently lead to ineffective doses being administered. Additionally, difficulties exist with oral administration of medications in this species, including the animals spitting out the drug [3]. This may again lead to failure of dosing or underdosing.

In this case, oral triclabendazole administered at a dose of 15 mg/kg was ineffective in resolving the alpaca’s symptoms. It is likely that the damage to hepatic parenchyma was too severe to be resolved, and secondary processes such as peritonitis, of which there was evidence at post-mortem examination, had already begun.

Hepatobiliary GGT was markedly increased and hepatocellular GLDH moderately increased in this alpaca, along with moderate anaemia and marked inflammatory changes in the leucogram, especially eosinophilia, with marked hyperglobulinemia. Interestingly, AST was not increased in this alpaca compared to other species [19]. Increases in hepatobiliary GGT and hepatocellular GLDH are highly tissue-specific and cannot be attributed to any other organ or system, and are long-known [19, 20] to be the most sensitive and specific biochemistry biomarkers for fasciolosis across species. A combination of the above biomarkers may be nearly pathognomonic for fasciolosis [21, 22]. Collection of blood samples for haematological and biochemical analysis can easily be carried out in the field, and results returned quickly and with relatively little expense, even when an external laboratory is used. For these reasons, the authors recommend this as a first-line diagnostic test in cases where acute fasciolosis is suspected. It must be noted however that, as demonstrated in this report, these parameters can change rapidly, and the combination of biomarkers described may not always be present in a single blood sample from an animal with acute fasciolosis.

In Ireland, alpacas are currently not classed as a food-producing species, meaning that veterinary surgeons are not restricted in their choice of medicines by the legislation which governs their use in the food-producing species. Buprenorphine was administered intravenously at a dose of 0.01 mg/kg. This resulted in extreme dysphoria, with vocalisation and frenzied activity. This made it difficult to assess the degree of pain relief provided and the treatment was not repeated. The metabolism of buprenorphine occurs primarily in the liver, consistent with the other opioids [23]. In humans with severe hepatic impairment, buprenorphine exposure was significantly increased when administered sublingually in combination with naloxone, compared to healthy subjects [24]. This increase in exposure could increase the potential for adverse side-effects, and is one possible explanation for the observed dysphoria in this alpaca. Although a similar association between hepatic impairment and buprenorphine exposure has not been demonstrated in alpacas, dysphoria was noted with intravenous administration of a higher dose (0.02 mg/kg) of buprenorphine to healthy adult alpacas [25], supporting the idea that increased exposure can produce this effect. Buprenorphine has also been used successfully in alpacas without provoking dysphoric effects. Doses of 0.014 mg/kg intramuscularly following orthopaedic surgery in a 10-day-old cria [26], 0.06 μg/kg subcutaneously following exploratory laparotomy in an 18-month old alpaca [27] and 0.01 mg/kg intravenously every 8 h in a 9 year old alpaca with a mandibular fracture [28] were not reported to result in dysphoria. Overall, the evidence suggests that there may be a risk of dysphoric adverse effects when buprenorphine is used in alpacas at higher doses, and possibly in cases of hepatic insufficiency. Veterinary practitioners should be aware of the potential for this when choosing buprenorphine to provide pain relief in this species.

This case report is the first comprehensive description of a case of acute fasciolosis in an individual alpaca in the literature. It also describes, to our knowledge, the first case of fasciolosis in Irish alpacas. However, given the growing popularity of this species, we can expect an increase in the apparent prevalence of this disease. This makes dissemination of information on the presentation, diagnosis and treatment of this disease, as described in this report, to veterinary practitioners who may attend alpacas absolutely imperative. This case also presented rather unusually, with colic as the primary presenting sign. Again, to the authors’ knowledge, colic has not previously been reported as a clinical sign of acute fasciolosis. Differential diagnoses in this case included TCU, peritonitis, gastrointestinal obstruction, urethral obstruction and pain due to stretching of a non-digestive organ capsule. This case demonstrates that acute fasciolosis should be a differential diagnosis for any alpaca presenting with abdominal pain, particularly if liver enzyme activity and eosinophil count are also raised. Consequently, haematological and biochemical analysis is recommended as an accessible, relatively inexpensive first-line diagnostic test in cases where acute fasciolosis is suspected. The diagnosis of acute fasciolosis was made in spite of previous flukicide treatment, which could have failed due to resistance to the flukicides administered or underdosing due to lack of weighing facilities, difficulties with administration and concerns regarding albendazole toxicity. Future studies should investigate the possible presence of albendazole resistance in this species. When considering the symptomatic treatment of these cases, buprenorphine should be used with care, as there may be potential for increased exposure in alpacas with hepatic insufficiency, which could result in adverse side effects. Further research should be carried out into alternative analgesics which could be used in cases where there is a high risk of adverse side effects with the administration of buprenorphine. This case report has described the clinical presentation and progression of clinical signs in a case of acute fasciolosis in an alpaca, along with the results of a range of diagnostic tests. This information may assist veterinary practitioners, not only in Ireland but internationally, in the diagnosis of this disease.

## Data Availability

The datasets generated and/or analysed during the current study are not publicly available due to client confidentiality/GDPR but are available in anonymised form from the corresponding author on reasonable request.

## Abbreviations

AST: aspartate aminotransferase
C1: first stomach compartment of the pseudoruminant
C3: third stomach compartment of the pseudoruminant
GGT: gamma-glutamyltransferase
GLDH: glutamate dehydrogenase
TCU: third compartment ulceration
UCD: University College Dublin
ULN: upper limit of normal1
